# Supplementary material for: Improved Humoral Immunity and Protection against Influenza Virus Infection with a 3d Porous Biomaterial Vaccine
Source: Adv Sci (Weinh). 2023 Sep 26;10(31):2302248. doi: 10.1002/advs.202302248 (PMC10625058; doi:10.1002/advs.202302248)
Supplement: Supplementary file 1 — Supporting Information [file ADVS-10-2302248-s001.pdf]

ADVANCED SCIENCE
Open Access

Supporting Information

for *Adv. Sci.*, DOI 10.1002/advs.202302248

Improved Humoral Immunity and Protection against Influenza Virus Infection with a 3d Porous Biomaterial Vaccine

Hiromi Miwa, Olivia Q. Antao, Kindra M. Kelly-Scumpia, Sevana Baghdasarian, Daniel P. Mayer, Lily Shang, Gina M. Sanchez, Maani M. Archang, Philip O. Scumpia, Jason S Weinstein* and Dino Di Carlo**

SUPPORTING INFORMATION FOR:

Improved humoral immunity and protection against influenza virus infection with a 3D porous biomaterial vaccine

Hiromi Miwa#, Olivia Q Antao#, Kindra M. Kelly-Scumpia, Sevana Baghdasarian, Daniel P. Mayer, Lily Shang, Gina M. Sanchez, Maani M Archang, Philip O. Scumpia, Jason S Weinstein*, Dino Di Carlo**

*# co-first author, * co-corresponding author*

H. Miwa, S Baghdasarian, L Shang, M.M. Archang, D. Di Carlo,
Department of Bioengineering University of California Los Angeles, Los Angeles, CA 90095, USA
E-mail: dicarlo@ucla.edu

H. Miwa, S Baghdasarian, L Shang, M.M. Archang, D. Di Carlo,
Department of Bioengineering University of California Los Angeles, Los Angeles, CA 90095, USA
E-mail: dicarlo@ucla.edu

O.Q. Antao, D.P. Mayer, G.M. Sanchez, J.S. Weinstein
Center for Immunity and Inflammation, Rutgers New Jersey Medical School, Newark, NJ, 07103, USA
E-mail: jason.weinstein@rutgers.edu

K.M. Kelly-Scumpia
Division of Cardiology, Department of Medicine David Geffen School of Medicine University of California, Los Angeles, Los Angeles, CA 90095, USA

P.O. Scumpia
Division of Dermatology, Department of Medicine David Geffen School of Medicine University of California, Los Angeles, Los Angeles, CA 90095, USA
E-mail: PScumpia@mednet.ucla.edu

M.M. Archang,
MSTP Program, David Geffen School of Medicine, University of California Los Angeles, Los Angeles, CA 90095, USA

P.O. Scumpia
Department of Dermatology VA Greater Los Angeles Healthcare System Los Angeles, CA 90073, USA

D. Di Carlo
Department of Mechanical and Aerospace Engineering University of California, Los Angeles Los Angeles, CA 90095, USA

D. Di Carlo
California Nano Systems Institute (CNSI) University of California, Los Angeles Los Angeles, CA 90095, USA

P.O. Scumpia, D. Di Carlo
Jonsson Comprehensive Cancer Center University of California, Los Angeles Los Angeles, CA 90095, USA

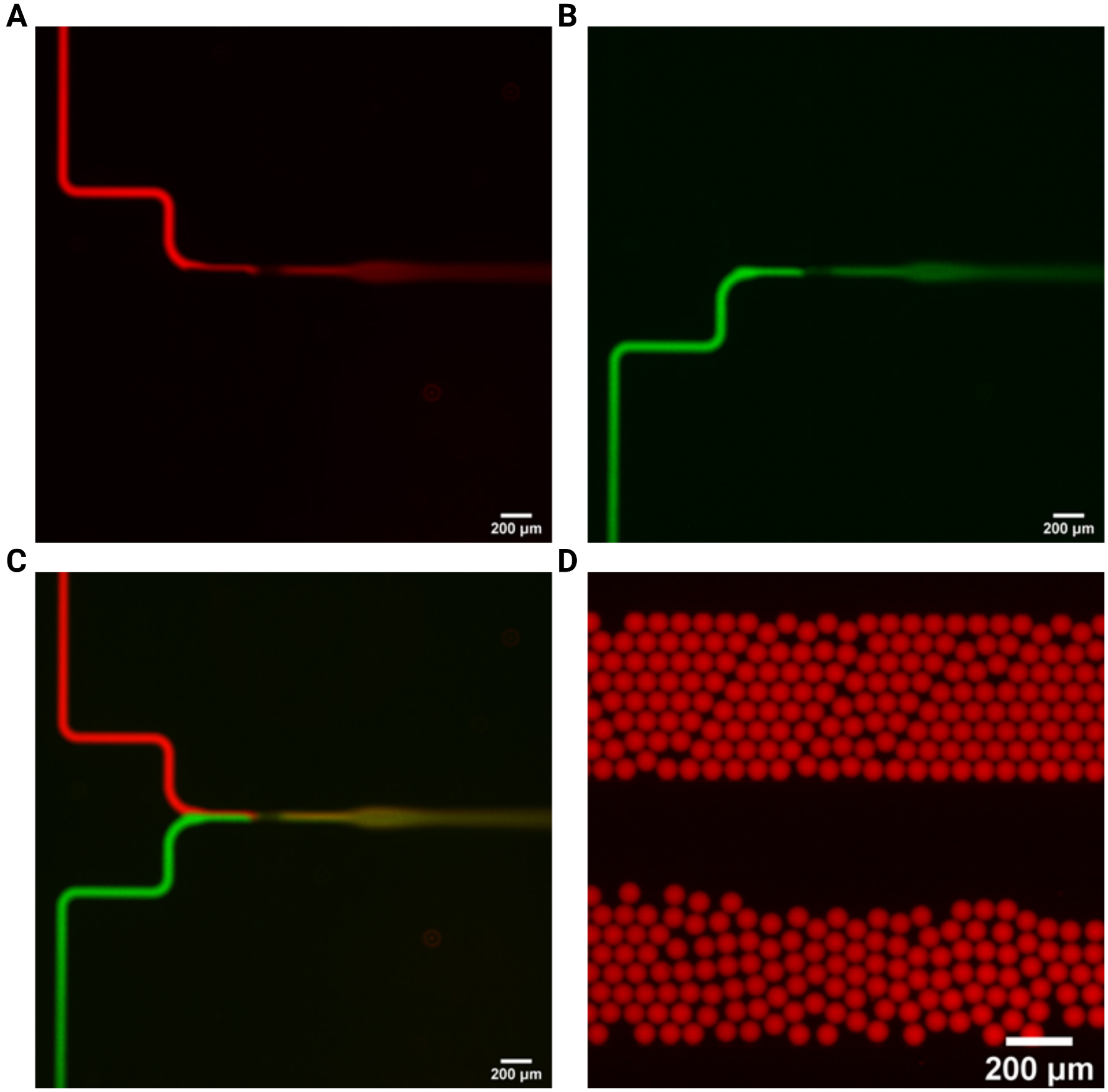

Figure S1. Microfluidics VaxMAP fabrication. Channels are highlighted with fluorescent dye solutions. Homogeneous droplets containing pregel solution and crosslinker form at a flow focusing junction of the microfluidic channel. A) The aqueous inlet channel with fluorescently tagged OVA (green) contains 4-arm PEG-vinylsulfone pregel solution. B) The second aqueous channel contains AlexaFluor 546-maleimide (red) with MMP-sensitive cross linker solution. C) Merged channel image. D) Fluorescence images of droplets generated downstream. Scale bars are 200 µm.

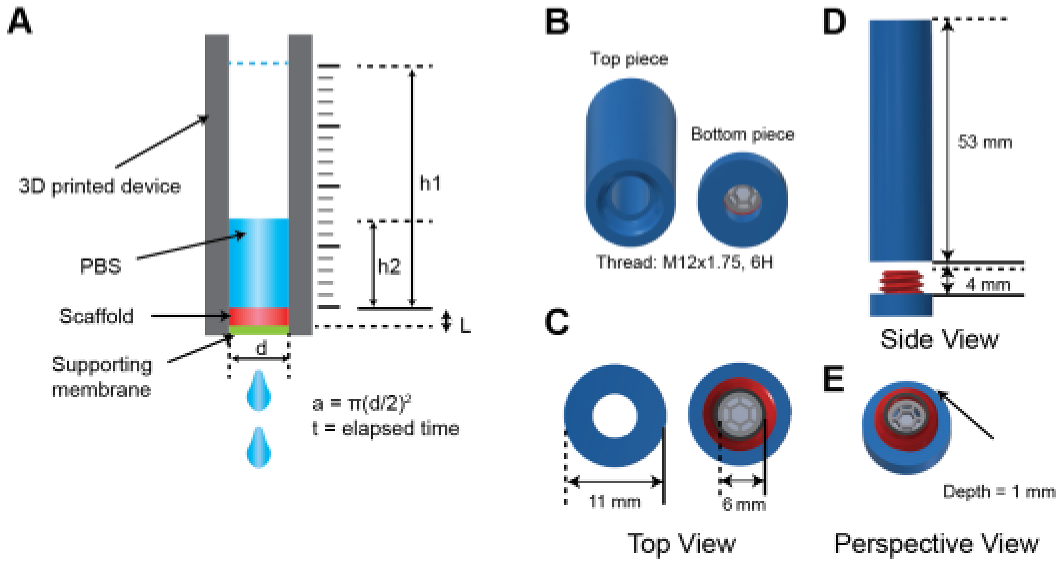

Figure S2. Hydraulic conductivity measurement apparatus. A) Schematic of the hydraulic conductivity measurement using a 3D-printed device. The initial height (h1) and final height (h2) of PBS that flows through the scaffold over an elapsed time were recorded to calculate the overall volumetric flow rate and the conductivity. B) Two components of the 3D-printed device viewed from C) top, D) side and E) perspective.

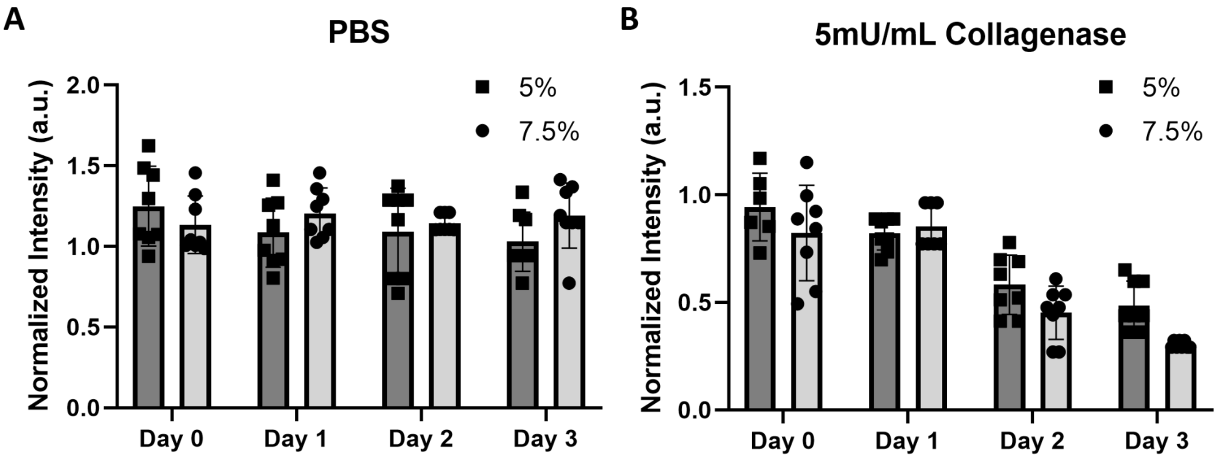

Figure S3. Microgels encapsulated with Fluorescent OVA antigen are imaged daily for 3 days. A) The fluorescence intensity remains stable in PBS. B) The fluorescence intensity gradually decreases in the collagenase solution as the antigen is being released.

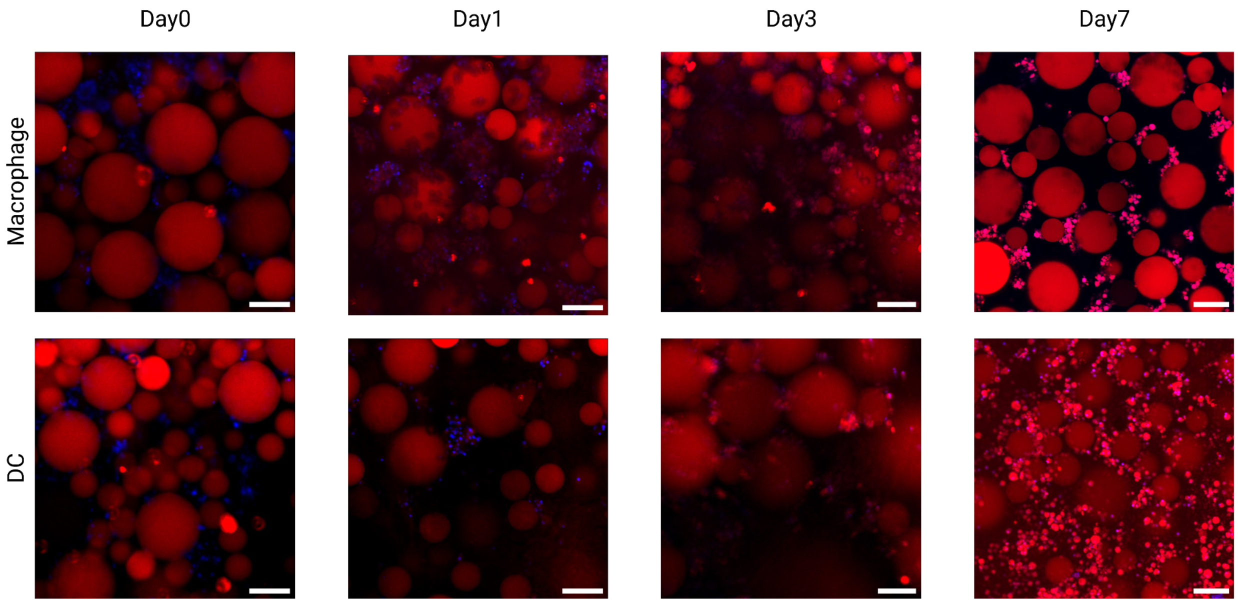

Figure S4. In vitro culture of mouse bone marrow derived macrophages and dendritic cells (DCs). Cells are cultured within 3D MAP scaffolds without antigen over 7 days. As the incubation period increased, MAP-derived fluorescence signal (Alexa Fluor 555-maleimide) accumulated in the cells. Blue: DAPI stain, Red: Alexa Fluor 555. Scale bars are 100 μm.

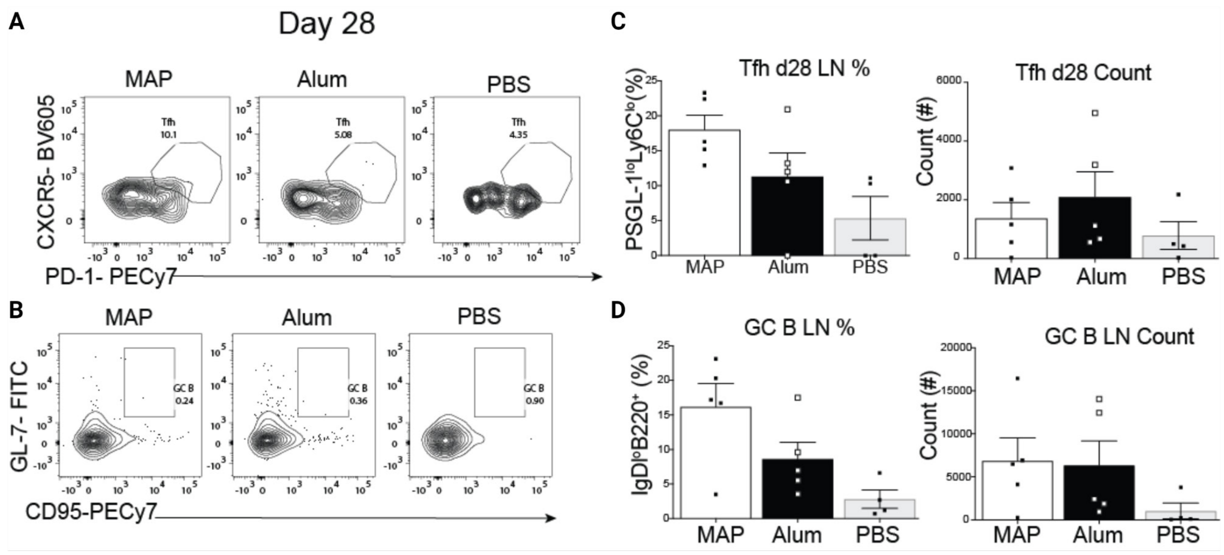

Figure S5. Tfh and GC B cell responses at day 28 post immunization from 5% VaxMAP injection. A-B) Representative FACS plots, percentages and counts of Tfh cells. C-D) Representative FACS plots, percentages and counts of GC B cells.

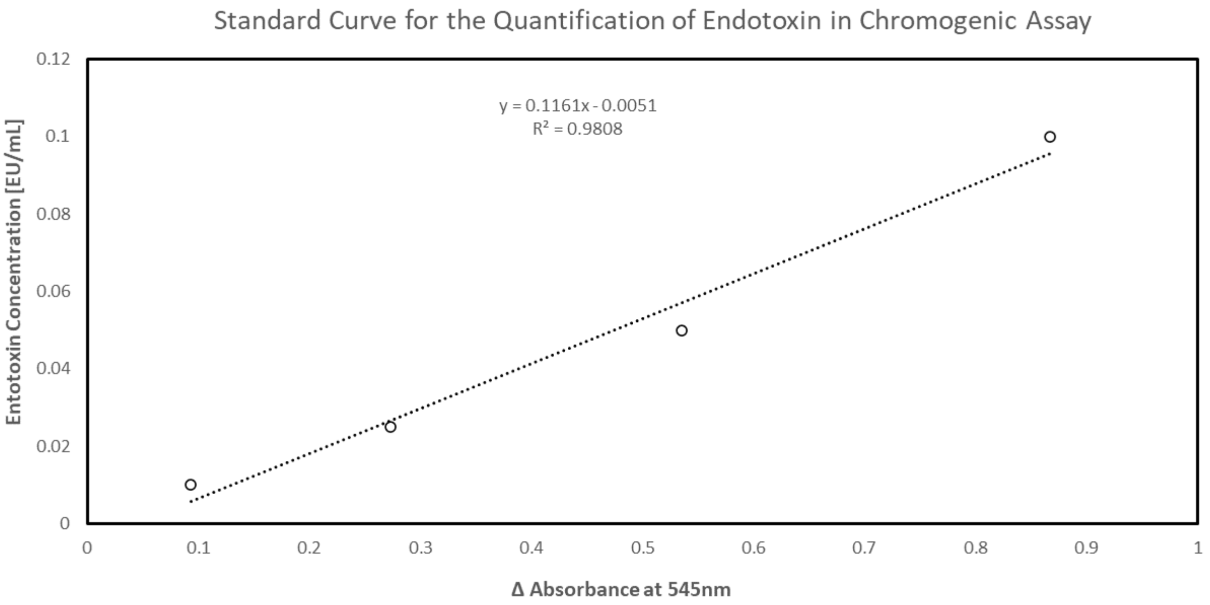

Figure S6. Endotoxin assay. VaxMAP is tested for concentration of endotoxin using an assay kit (Genscript ToxinSensor™ Chromogenic LAL Endotoxin Assay Kit). Fabricated VaxMAPs are mixed with ToxinSensor reagents and absorbance at 545nm is measured to confirm endotoxin concentration was less than 0.01 EU/mL before animal testing. Standard curve is shown to calibrate threshold levels.

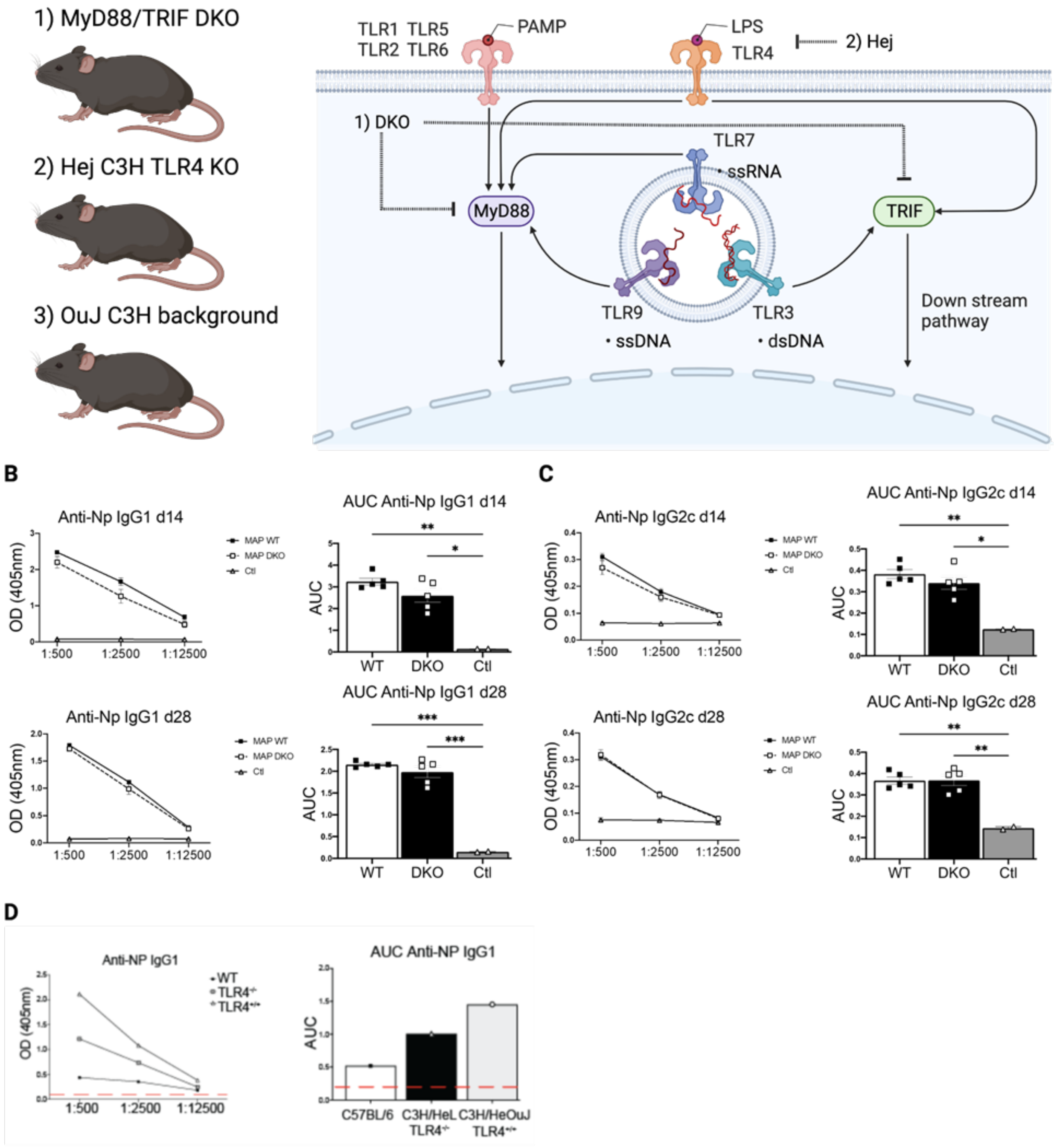

Figure S7. MyD88/TRIF DKO. A) Schematic of signaling pathway of importance in innate immunity. B) Optical Density and Area Under the Curve of anti-NP IgG1 antibodies at day 14 (top) or 28 (bottom) post immunization. C, D) Optical Density and Area Under the Curve of anti-NP IgG2c antibodies at day 14 (top) or 28 (bottom) post immunization.

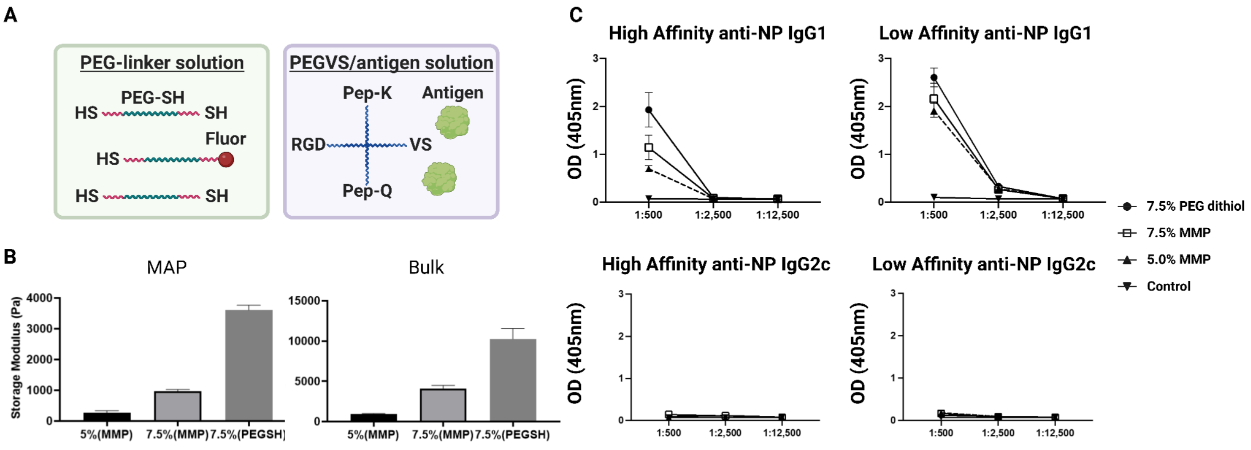

Figure S8. Effect of MAP gel formulation on high and low affinity antibody titers. A) MAP microgels produced with 4-arm PEG-vinylsulfone (PEG-VS) via thiol-ene reactions to encapsulate antigen in the dense gel mesh. PEG dithiol was used as a crosslinker to enable microparticle formulations with higher stiffness than MMP crosslinkers. B) Stiffness of PEG dithiol crosslinked gels formed in bulk and annealed MAP gel form. PEG dithiol crosslinking resulted in more than 2-fold increased stiffness than the 7.5% MMP crosslinked condition. C) Optical density of high affinity (NP-9) and low affinity (NP-27) anti-NP IgG1 and IgG2c antibodies at 14 days post immunization. PEG represents PEG dithiol crosslinked condition. PEG dithiol was crosslinked with 7.5% of PEG-VS. 7.5% and 5% MAP represented MMP crosslinked conditions.

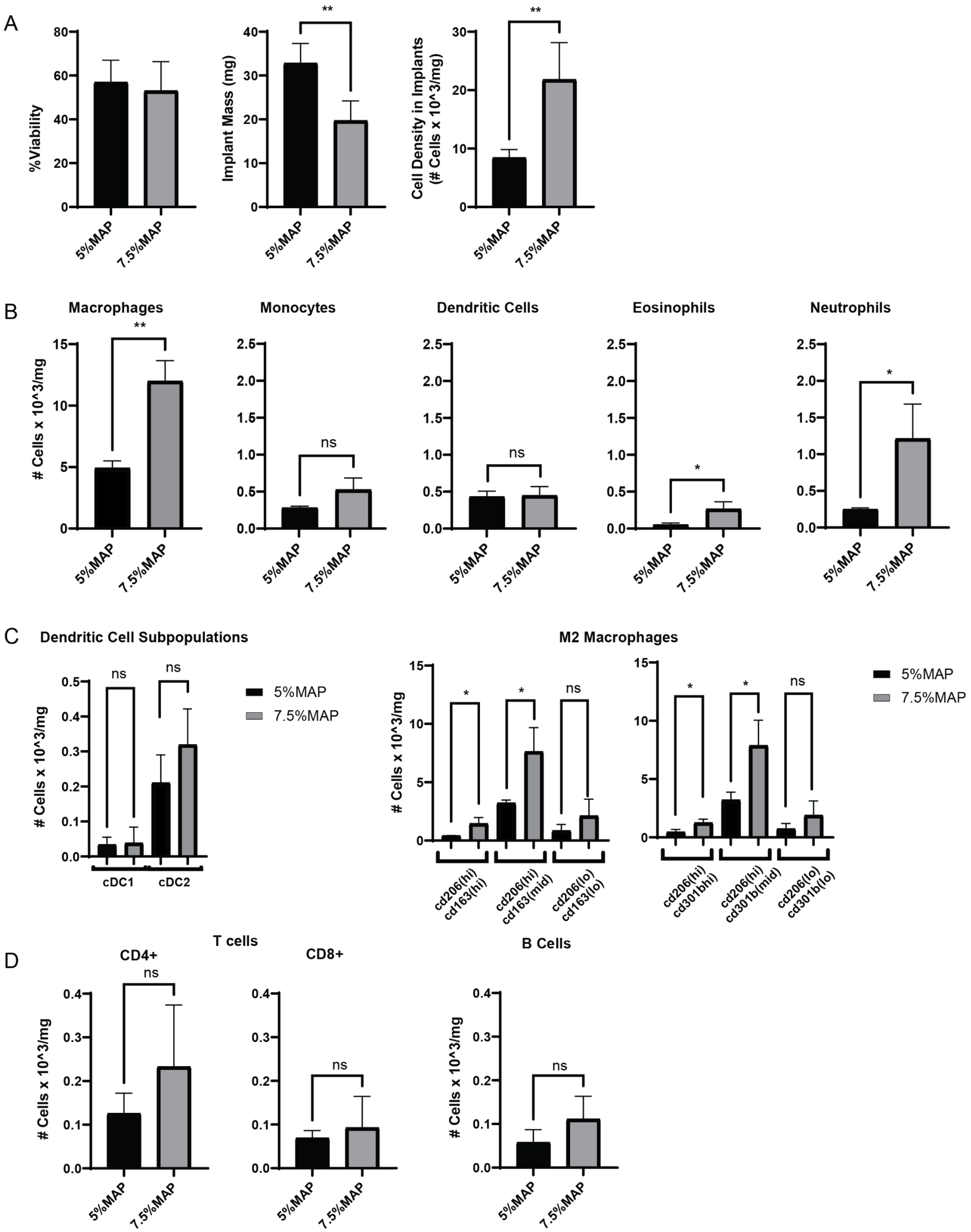
A
%Viability
Implant Mass (mg)
Cell Density in Implants (# Cells x 10^3/mg)
**
5%MAP
7.5%MAP
B
Macrophages
Monocytes
Dendritic Cells
Eosinophils
Neutrophils
Cells x 10^3/mg
ns
*
C
Dendritic Cell Subpopulations
cDC1
cDC2
M2 Macrophages
cd206(hi) cd163(hi)
cd206(hi) cd163(mid)
cd206(lo) cd163(lo)
cd206(hi) cd301bhi)
cd206(hi) cd301b(mid)
cd206(lo) cd301b(lo)
D
T cells
CD4+
CD8+
B Cells

Figure S9. Effect of crosslinking density (independent of antigen release) on immune cell activation. A) Cell viability, weighted mass of the implants, and cell density of collected implants 7 days post implantation. B) Cell numbers per implant were obtained by flow cytometry of various myeloid cell populations including total macrophages (F4/80$^+$CD11b$^+$), monocytes (Ly6C$^+$CD11b$^+$), dendritic cells (DC, CD11c$^+$MHCII$^+$), neutrophils (Siglec-F$^-$Ly6G$^+$CD11b$^+$), and eosinophils (Siglec-F$^+$Ly6G$^-$CD11b$^+$) seven days after implantation. C) Left panel shows conventional (c)DC1 (CD8α+CD11b-CD11c+MHCII+ and cDC2 (CD11b$^+$SIRPα$^+$CD11c$^+$MHCII$^+$) subpopulations of DCs and right panel denotes levels of M2 macrophage marker (CD206 and CD163 or CD301b and CD206) expression of all F4/80$^+$CD11b$^+$ macrophages in terms of density of cells per implant as all macrophages within 5% and 7.5% MAP. All macrophages displayed expression of at least one of these M2 macrophage markers. D) T (CD3$^+$CD4$^+$ or CD3$^+$CD8$^+$) and B cell populations (B220$^+$CD19$^+$) in terms of density of cells per implant. Representative plots for stains are shown in Figure S9. * represents $P < 0.05$. ** represents $P < 0.01$.

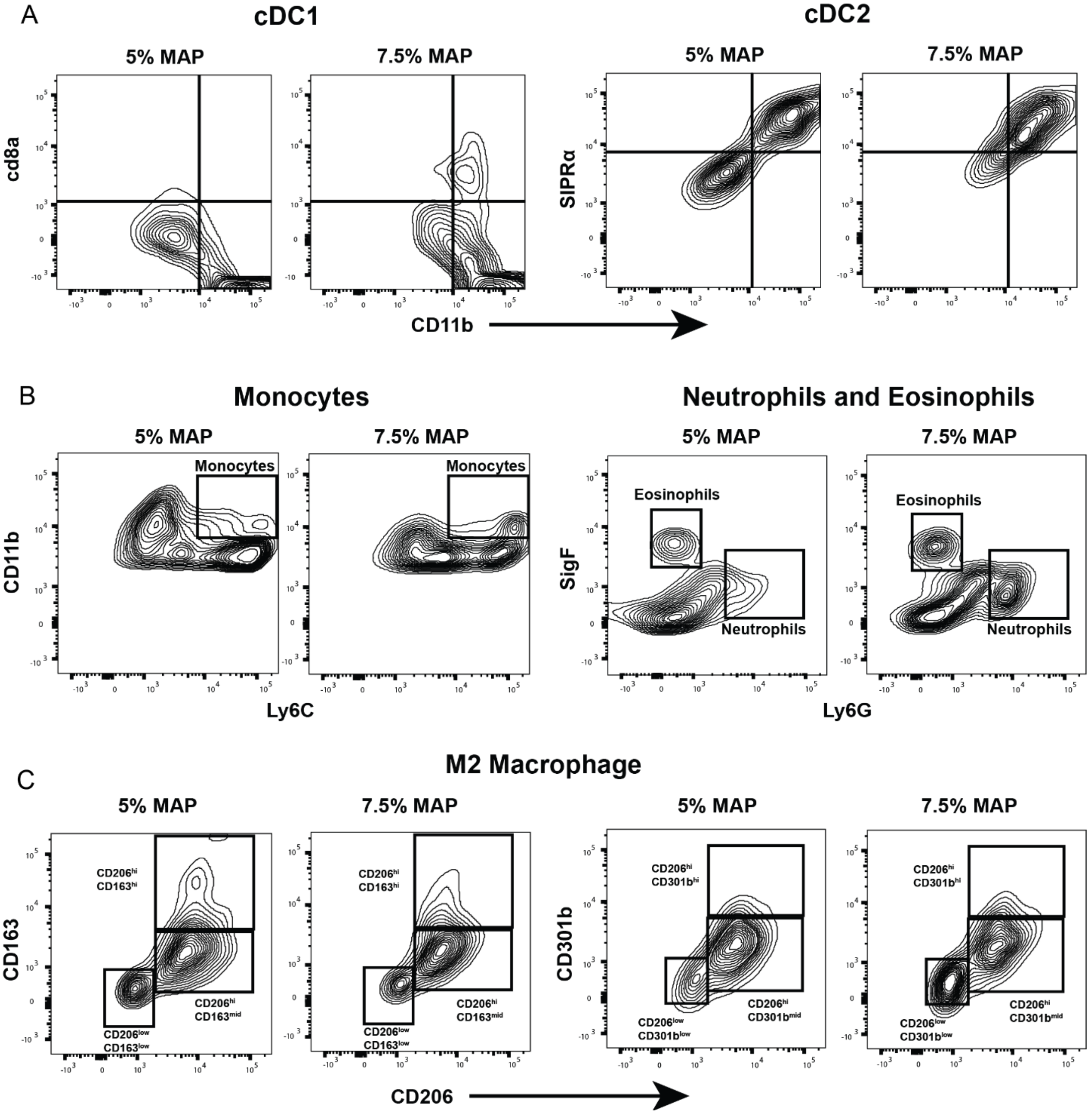

Figure S10. Representative FACS plots for myeloid cell populations. For all cell populations other than macrophages, F4/80+ cells were excluded/gated out prior to examining the population of interest to eliminate highly autofluorescent macrophages. A) cDC1 and cDC2 plots following gating on $CD11c^{+}MHCII^{+}$ cells. The cDC1 population is defined as $CD11c^{+}MHCII^{+}CD8\alpha^{+}CD11b^{-}$ (upper left quadrant). The cDC2 population is defined as $CD11c^{+}MHCII^{+}SIPR\alpha^{+}CD11b^{+}$ (upper right quadrant). B) Representative FACS plots of monocytes, neutrophils, and eosinophils. The monocyte population is defined as $CD11b^{+}Ly6C^{+}$

cells. The neutrophil population is defined as $CD11b^+Ly6G^+Siglec\text{-}F^-$ cells. The eosinophil population is defined as $CD11b^+SiglecF^+Ly6G^-$ cells. C) Representative FACS plots of the M2 macrophage populations. The macrophage population is defined as $CD11b^+F4/80^+$ cells and was separated based on CD163 and CD206 expression or CD301b and CD206 expression.

Table S1. Antibodies Used for Flow Cytometry

Antigen	Dilution	Clone	Fluorochrome	Source	Cat#
CD4	1:200	RM4-5	APC	Biolegend	100516
CD4	1:200	RM4-5	AF700	Biolegend	
CD44	1:200	IM7	APC Cy7	Biolegend	103028
B220	1:200	RA3-6B2	BV605	Biolegend	103244
IgD	1:200	11-26c.2a	BV421	Biolegend	405225
GL-7	1:200	GL-7	Alexa488	Biolegend	144612
CD95	1:200	Jo2	PE Cy7	BD Pharmagen	557653
PSGL-1	1:1000	2PH1	Pacific Blue	BD Biosciences	562807
Ly6C	1:400	HK1.4	BV510	Biolegend	128033
PD-1	1:200	29F.1A12	PE-Cy7	Biolegend	135216

CXCR5	1:100	L138D7	BV605	Biolegend	145513

Table S2. Chemicals and Peptides

Reagent or Resource	Source	Cat #
Phosphate Buffered Saline (PBS)	Sigma	806544
RPMI-1640	Corning	10-040-CM
Fetal Bovine Serum	VWR	89510-186

Table S3. Antibodies used for MAP Implant Flow Cytometry

Macrophage Panel			
**Antigen**	**Fluorochrome**	**Dilution**	**Source**
CD11b	PE	1:200	Biolegend
F4/80	PerCP-Cy 5.5	1:200	eBiosciences
Dendritic Cell Panel			
**Antigen**	**Fluorochrome**	**Dilution**	**Source**
CD11b	PE-Cy7	1:200	Biolegend
CD11c	PerCP	1:200	Biolegend
MHCII	FITC	1:200	Biolegend
F4/80	PE	1:200	eBiosciences
CD8a	APC-Cy7	1:200	Biolegend
SIRPα	APC	1:200	Biolegend

Neutrophils/Monocytes/Eosinophils Panel			
**Antigen**	**Fluorochrome**	**Dilution**	**Source**
CD11b	FITC	1:200	Biolegend
F4/80	APC	1:200	Biolegend
Gr1	APC-Cy7	1:200	Biolegend
Ly6C	PerCP-Cy 5.5	1:200	eBiosciences
Ly6G	PE-Cy7	1:200	Biolegend
Siglec-F+	PE	1:200	eBiosciences
T cells/B cells Panel			
**Antigen**	**Fluorochrome**	**Dilution**	**Source**
CD3	FITC	1:200	Biolegend
CD4	PerCP	1:200	Biolegend
CD8a	PE-Cy7	1:200	Biolegend

B220	APC	1:200	eBiosciences
CD19	APC-Cy7	1:200	eBiosciences
CD11b	PE	1:200	Biolegend
F4/80	PE	1:200	eBiosciences
